# Preoperative Geriatric Nutritional Risk Index (GNRI) and Comorbidity Burden as Mortality Risk Markers After Proximal Femoral Nailing in Older Patients with Pertrochanteric Hip Fractures

**DOI:** 10.3390/jcm15145400

**Published:** 2026-07-09

**Authors:** Mehmet Burak Gökgöz, Hamit Çağlayan Kahraman, Volkan Gür, Akın Öztürk, İbrahim Doğan, Alper Gönbe, Berat Avcı, Nizamettin Koçkara, Hakan Sofu, Furkan Yapıcı

**Affiliations:** 1 Department of Orthopedics and Traumatology, Faculty of Medicine, Erzincan Binali Yıldırım University, 24100 Erzincan, Turkey; dr.m.burakgokgoz@gmail.com (M.B.G.); drvolkangur@hotmail.com (V.G.); akin-ozturk@hotmail.com (A.Ö.); ibrahimdogan2424@gmail.com (İ.D.); alpergonbe@yandex.com (A.G.); dr.braa90@gmail.com (B.A.); nzmttn@yahoo.com (N.K.); 2 Department of Orthopedics and Traumatology, Fatih Sultan Mehmet Training and Research Hospital, University of Health Sciences, 34372 İstanbul, Turkey; drhcaglayan@gmail.com; 3 Department of Orthopedics and Traumatology, Faculty of Medicine, Istanbul Nişantaşı University, 34398 İstanbul, Turkey; hakansofu@yahoo.com

**Keywords:** hip fracture, pertrochanteric fracture, proximal femoral nailing, Geriatric Nutritional Risk Index, nutritional risk, Charlson comorbidity burden, neutrophil-to-lymphocyte ratio, systemic immune-inflammation index, mortality, orthogeriatrics

## Abstract

**Background:** Mortality after geriatric hip-fracture surgery remains substantial. This study evaluated whether preoperative GNRI, comorbidity burden, and CBC-derived inflammatory indices were associated with one-year and long-term mortality after PFN for pertrochanteric/intertrochanteric fractures. **Methods:** In this single-centre retrospective cohort study of prognostic risk markers, 248 PFN records were screened. After excluding six incomplete records and 25 patients aged <65 years, 217 older fracture/surgical episodes formed the time-to-event cohort; 194 were evaluable for binary one-year mortality. Living patients/episodes with <365 days of follow-up were censored in survival analyses. Logistic and Cox models were supported by Firth penalized logistic regression, calibration assessment, and internal validation. **Results:** Median age was 82 years, 65.0% were female, and 68.7% were ASA III–IV. Overall, 96/217 (44.2%) died during median follow-up of 570 days. Thirty-day, 90-day, and one-year evaluable mortality were 7.4%, 15.2%, and 27.3%. GNRI < 82 identified a small high-risk subgroup: 10/13 evaluable patients/episodes (76.9%) died within one year versus 43/181 (23.8%) with GNRI ≥ 82. GNRI < 82 remained associated with one-year mortality in adjusted logistic regression, although with a wide confidence interval due to sparse subgroup size (OR 6.43, 95% CI 1.50–27.55; *p* = 0.012), and in Firth penalized sensitivity analysis (OR 5.51, 95% CI 1.34–22.61; *p* = 0.018). In Cox analysis, age, ASA III–IV, available Charlson-domain comorbidity burden, and lower continuous GNRI were associated with long-term mortality, whereas adding GNRI < 82 and NLR did not materially improve cross-validated discrimination. **Conclusions:** GNRI < 82 identified a small subgroup with high observed mortality after PFN. Because the subgroup was sparse and biomarker addition did not materially improve internally validated discrimination, GNRI should be treated as an alerting clinical flag rather than a stand-alone basis for patient-level risk prediction. External validation is required.

## 1. Introduction

Hip fractures in older adults represent a sentinel event of frailty, multimorbidity, and functional decline. They are associated with substantial early and long-term excess mortality, particularly during the first postoperative months, and the survival penalty may persist beyond the acute episode [1,2,3]. Pertrochanteric and intertrochanteric fractures are commonly treated with intramedullary fixation using a proximal femoral nail (PFN), yet postoperative survival is strongly influenced by physiological reserve, perioperative systems of care, and medical vulnerability rather than implant-related factors alone [4,5,6].

Conventional perioperative risk assessment in hip-fracture patients relies heavily on age, ASA physical status, and comorbidity burden. ASA physical status has demonstrated prognostic value in large hip-fracture datasets, and the Charlson Comorbidity Index remains a widely used approach to quantifying multimorbidity and mortality risk [7,8]. However, hip fracture in older adults is also an acute biological stressor superimposed on malnutrition, sarcopenia, inflammation, cognitive impairment, and reduced rehabilitation capacity; therefore, routinely available laboratory and anthropometric indices may add clinically relevant information beyond traditional risk scores.

The Geriatric Nutritional Risk Index (GNRI), derived from serum albumin and the ratio of actual to ideal body weight, was originally developed as an objective indicator of nutrition-related risk in hospitalized older adults [9]. The recent hip-fracture literature has strengthened the evidence that malnutrition and low GNRI are associated with postoperative complications and mortality, including systematic reviews/meta-analyses and cohort studies addressing 30-day, 180-day, one-year, and longer-term outcomes [10,11,12,13,14,15,16,17].

In parallel, complete blood count-derived inflammatory indices, including the neutrophil-to-lymphocyte ratio (NLR), platelet-to-lymphocyte ratio (PLR), and systemic immune-inflammation index (SII), have been investigated as accessible markers of perioperative inflammatory response after hip fracture [18,19,20,21,22]. Increasingly, hip fracture in older adults is understood as a systemic geriatric event rather than an isolated orthopedic injury, with outcomes shaped by frailty, sarcopenia, cognitive impairment, nutritional reserve, and rehabilitation and care-pathway factors [23,24]. In design terms, this was a single-centre retrospective cohort study using institutional PFN records and registry-based mortality follow-up. The present study evaluated preoperative GNRI, comorbidity burden, and complete blood count-derived inflammatory indices after PFN in older patients with pertrochanteric/intertrochanteric hip fractures, with explicit separation of one-year binary mortality from long-term time-to-event mortality and internal assessment of model behaviour.

## 2. Materials and Methods

### 2.1. Study Design and Reporting Framework

This was a retrospective, single-centre cohort study of prognostic risk markers conducted at Erzincan University School of Medicine Mengucek Gazi Training and Research Hospital. Consecutive PFN records for pertrochanteric/intertrochanteric femoral fractures performed between 4 September 2015 and 26 September 2025 were screened. The surgical screening period and administrative follow-up were distinct: case accrual ended on 26 September 2025, whereas vital-status follow-up for living patients/episodes continued until administrative censoring on 19 February 2026. Reporting followed STROBE principles for observational studies, and regression-model reporting was TRIPOD-informed because logistic and Cox models were used to describe model behaviour and internal validity [25,26,27]. The study was conducted in accordance with the Declaration of Helsinki. The ethics committee approval and permission for the study were obtained from Erzincan University Faculty of Medicine Ethics Committee with the decision number 2026-04/04 and date 19 February 2026. No formal sample-size calculation was performed; the study size was determined by the number of eligible complete records in the institutional dataset.

### 2.2. Study Population and Analytic Cohorts

According to the institutional PFN screening log, 248 source PFN records were identified. Six records lacked the complete core laboratory or anthropometric data required for GNRI and inflammatory-index calculation, leaving 242 complete adult analytic records. Patients were eligible for the geriatric analytic cohort if they were aged ≥65 years and underwent PFN for an acute pertrochanteric or intertrochanteric femoral fracture. All included procedures were performed using the Talon DistalFix proximal femoral nail system (Oliga Machinery and Electronics Import-Export Limited Company, Ankara, Türkiye), with implant dimensions selected according to fracture morphology, patient anatomy, and surgeon preference. The analytic file did not contain consistently complete AO/OTA or Evans fracture subclassification; therefore, the cohort was defined by the anatomic pertrochanteric/intertrochanteric diagnosis and PFN treatment, and fracture subclassification was not entered as an adjustment variable. According to the screening log, patients with pathological fractures, periprosthetic fractures, high-energy polytrauma, revision fixation, or nonoperative management were not included in the source cohort. Because the clinical question concerned geriatric hip-fracture mortality, the primary time-to-event cohort was restricted to patients aged ≥65 years, yielding 217 eligible surgical/fracture episodes. For the binary one-year mortality analysis, living patients/episodes with less than 365 days of follow-up were not classified as one-year survivors; instead, they were excluded from the binary one-year model and retained as right-censored observations in Cox survival analysis. This approach avoided misclassifying 2025 cases without a full 365-day outcome opportunity as one-year survivors, while allowing Cox models to use their available follow-up time up to censoring. This yielded 194 patients/episodes evaluable for the primary one-year binary endpoint. Administrative censoring for living patients/episodes was performed using the dataset lock date of 19 February 2026. The primary unit of analysis was the eligible surgical/fracture episode. In the complete analytic dataset, two identifiers contributed two eligible PFN episodes each; therefore, a first-episode-only sensitivity analysis was performed to assess whether repeated episodes influenced the primary findings.

### 2.3. Data Collection and Index Calculation

Demographic data, ASA physical status, operative date, vital status, time from operation to death, anthropometric measures, complete blood count parameters, serum albumin, and recorded comorbidities were extracted from the institutional dataset. Height and weight were retrieved from routinely recorded institutional clinical fields; the retrospective dataset did not reliably distinguish directly measured values from estimated, rounded, or previously recorded anthropometric values. The following indices were calculated from preoperative values: NLR = neutrophil count/lymphocyte count; PLR = platelet count/lymphocyte count; SII = platelet count × neutrophil count/lymphocyte count; and GNRI = 1.489 × albumin (g/L) + 41.7 × min (actual body weight/ideal body weight, 1). The actual-to-ideal body weight ratio was capped at 1. Ideal body weight was calculated using the sex-specific Lorentz formula specified in the original GNRI definition: for men, height (cm) − 100 − [(height − 150)/4]; for women, height (cm) − 100 − [(height − 150)/2.5]. GNRI categories were prespecified as no risk (≥98), low risk (92 to <98), moderate risk (82 to <92), and high risk (<82) [9]. Preoperative laboratory values were extracted from routinely recorded preoperative laboratory fields in the institutional dataset. The analytic file did not reliably preserve whether these values represented admission samples, immediately preoperative samples, or the closest repeat value before surgery for all patients; therefore, they were analyzed as routinely available preoperative values rather than as precisely time-stamped biomarkers.

The recorded institutional comorbidity field represented an age-adjusted Charlson Comorbidity Index (ACCI). Because ACCI incorporates an age component by construction, it was not used as the primary comorbidity covariate in models that already included age. Instead, an available Charlson-domain weighted comorbidity-burden score was reconstructed from the comorbidity fields present in the dataset using original Charlson-compatible weights where mapping was possible [8]. This variable should be interpreted as an available-domain weighted burden score rather than a fully validated Charlson Comorbidity Index, because several Charlson domains and severity distinctions were not captured with complete granularity. This reconstructed score was used as an age-separated comorbidity-burden covariate and should not be interpreted as a validated Charlson Comorbidity Index. The mapping is provided in Supplementary Table S1. The recorded ACCI field was retained only for descriptive, correlation, sensitivity, and discrimination analyses. We did not assess internal consistency using Cronbach’s alpha because Charlson-domain comorbidities constitute a formative prognostic burden index rather than a reflective psychometric scale; individual comorbidities are not expected to be highly intercorrelated.

### 2.4. Outcomes

The primary endpoint was one-year all-cause mortality. Patients who died within 365 days of PFN were classified as one-year deaths; patients who died after 365 days or were alive with at least 365 days of follow-up were classified as one-year survivors for the binary analysis. Living patients/episodes with less than 365 days of follow-up were excluded from the binary one-year analysis and censored at last available follow-up in survival analyses. Secondary outcomes included 30-day and 90-day all-cause mortality and long-term all-cause mortality. Vital status and date of death were ascertained from hospital mortality registry and national death registry, and the dataset was locked on 19 February 2026 for administrative censoring.

### 2.5. Statistical Analysis

Continuous variables are presented as median and interquartile range (IQR), and categorical variables as n (%). Between-group comparisons for one-year mortality used the Mann–Whitney U test for continuous variables and chi-square or Fisher exact tests for categorical variables, as appropriate. One-year mortality was analyzed using multivariable logistic regression. Long-term mortality was analyzed using Cox proportional hazards regression. Prespecified covariates were selected on clinical grounds and included age, sex, ASA III–IV, available Charlson-domain weighted comorbidity burden, NLR, and GNRI. NLR was selected as the prespecified CBC-derived inflammatory covariate because it is the most commonly studied and clinically interpretable leukocyte-based inflammatory ratio in the hip-fracture mortality literature, whereas PLR and SII were evaluated as exploratory discriminatory markers to avoid overfitting the multivariable models with multiple correlated CBC-derived indices. Albumin was not entered into the same multivariable model as GNRI to avoid collinearity because albumin is part of the GNRI formula. For the primary one-year logistic model, the events-per-variable ratio was approximately 8.8 (53 events, 6 covariates). Although this model-level ratio was acceptable for exploratory adjustment, the GNRI < 82 exposure category was sparse; therefore, precision limitations were anticipated and adjusted estimates for this subgroup were interpreted cautiously. Discrimination and calibration analyses were performed to describe model behaviour and internal validity. Dementia/Alzheimer disease and individual comorbidities were not entered separately into the primary multivariable models because of the modest number of one-year events, potential overlap with the reconstructed Charlson-domain burden score, and the prespecified aim of maintaining an events-per-variable-conscious model.

Because GNRI < 82 represented a small high-risk subgroup, the primary logistic model was repeated using Firth penalized logistic regression as a sparse-data sensitivity analysis [28]. Prespecified sensitivity analyses included alternative GNRI operationalisations as a continuous variable and as nutritional-risk categories, Cox models using GNRI < 82 and GNRI categories, comparison of one-year evaluable patients/episodes with living patients/episodes censored before one year, Kaplan–Meier-estimated one-year mortality in the full time-to-event cohort, a full-opportunity cohort restricted to patients/episodes operated on at least 365 days before dataset lock, calendar-year sensitivity using operation year, correlation and sensitivity analyses for recorded ACCI versus the available Charlson-domain score, and a first-episode-only sensitivity analysis for repeated identifiers. For Cox models, proportional hazards assumptions were evaluated using Schoenfeld residuals and their association with log-transformed follow-up time [29]. Predictive discrimination for one-year mortality was assessed using apparent AUC with 1000 non-parametric bootstrap resamples for 95% confidence intervals. Internal validation included five-fold cross-validation stratified by one-year mortality status and bootstrap optimism-corrected AUCs. Cross-validated AUCs were calculated from pooled out-of-fold predictions. Bootstrap optimism-corrected AUCs were calculated from bootstrap model refitting. Calibration was evaluated using Brier score, Hosmer–Lemeshow testing, calibration plot, and cross-validated calibration intercept and slope calculated from pooled out-of-fold predicted probabilities. Statistical significance was set at two-sided *p* < 0.05. No formal multiplicity adjustment was applied; secondary, exploratory, and sensitivity analyses were interpreted as hypothesis-generating and supportive of the primary risk-marker framework. Analyses were performed in R (R Foundation for Statistical Computing, Vienna, Austria) and cross-checked in IBM SPSS Statistics for Windows, Version 31.0.1.0 (IBM Corp., Armonk, NY, USA). Descriptive analyses, group comparisons, standard logistic regression, Cox proportional hazards models, Kaplan–Meier analyses, and ROC analyses were reproduced in SPSS where applicable. Firth penalized logistic regression, bootstrap optimism correction, stratified five-fold cross-validation, calibration assessment, and proportional hazards diagnostics were implemented in R. Given the modest number of events and the sparse GNRI < 82 subgroup, multivariable estimates were interpreted as risk-marker associations rather than definitive causal effects.

## 3. Results

### 3.1. Cohort Derivation and Outcomes

**Figure 1:**
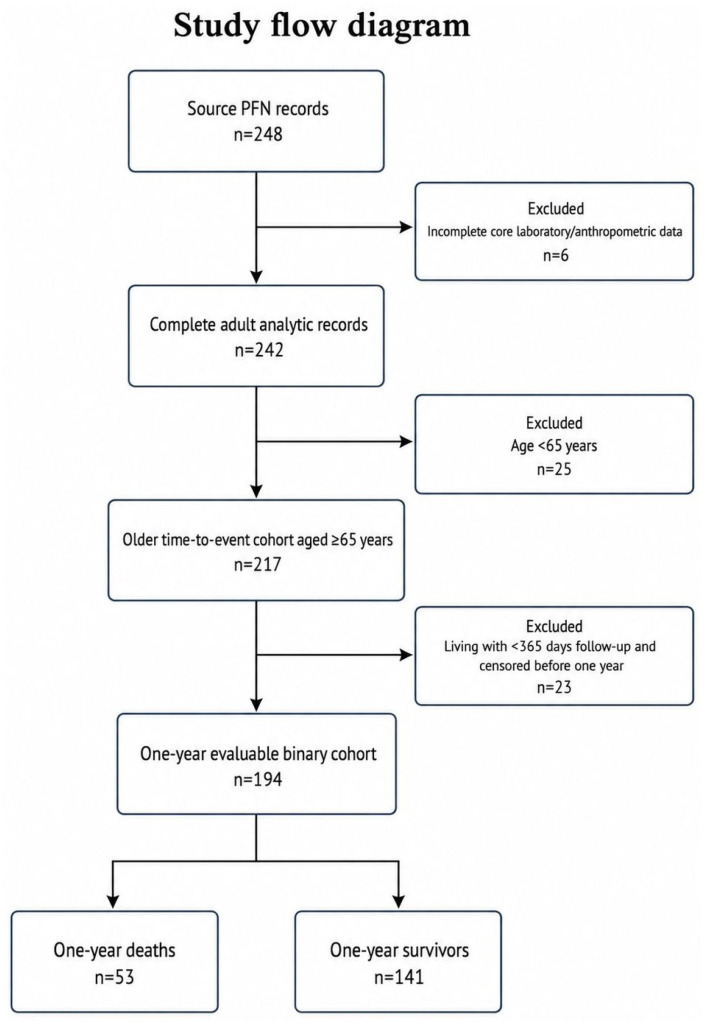
Patient/fracture-episode selection and analytic cohorts. Living patients/episodes with <365 days of follow-up were excluded from the binary one-year endpoint and retained as censored observations in Cox time-to-event analyses.

Of 248 source records, 242 had complete core data and 217 patients/episodes aged ≥65 years constituted the time-to-event cohort (Figure 1). The complete analytic records spanned operations performed from 4 September 2015 to 26 September 2025. In this older-adult cohort, median age was 82 years (IQR 74–86), 141 patients/episodes (65.0%) were female, and 149 (68.7%) were ASA III–IV. Overall, 96/217 (44.2%) died during a median follow-up of 570 days. Thirty-day and 90-day mortality were 16/217 (7.4%) and 33/217 (15.2%), respectively.

For the binary one-year mortality analysis, 194 patients/episodes were evaluable; 23 living patients/episodes had less than one year of follow-up and were treated as censored rather than one-year survivors. The 23 living patients/episodes censored before one year all underwent surgery in 2025. Baseline characteristics of one-year evaluable patients/episodes and living patients/episodes censored before one year were broadly similar, supporting the denominator strategy used for the binary endpoint (Supplementary Table S2). Among these 194 evaluable patients/episodes, 53 (27.3%) died within one year. Patients/episodes with one-year mortality were older, had lower albumin and GNRI, higher recorded ACCI and available Charlson-domain weighted burden, and were more frequently ASA III–IV (Table 1). NLR, PLR, and SII were numerically higher among one-year non-survivors, but these differences did not reach statistical significance. As a sensitivity analysis addressing incomplete one-year follow-up, the Kaplan–Meier-estimated one-year mortality was calculated in the full time-to-event cohort (n = 217). Estimated one-year mortality was 24.6% (95% CI, 18.9–30.4) overall, 71.4% (95% CI, 47.8–95.1) in the GNRI < 82 group, and 21.4% (95% CI, 15.7–27.1) in the GNRI ≥ 82 group, consistent with the binary one-year endpoint.

### 3.2. GNRI Categories and Early Mortality Signal

One-year mortality increased most clearly in the GNRI high-risk group. Among one-year evaluable patients, GNRI < 82 was present in 13 patients, of whom 10 died within one year (76.9%). By comparison, 43 of 181 patients with GNRI ≥ 82 died within one year (23.8%; unadjusted OR 10.70, 95% CI 2.82–40.64). Relative to the GNRI no-risk group, the unadjusted odds ratio for one-year mortality in the GNRI high-risk group was 11.94 (95% CI 2.83–50.42; *p* < 0.001; Table 2). Because this subgroup was small, this finding was interpreted as a high-risk signal and evaluated further using penalized logistic regression.

**Figure 2:**
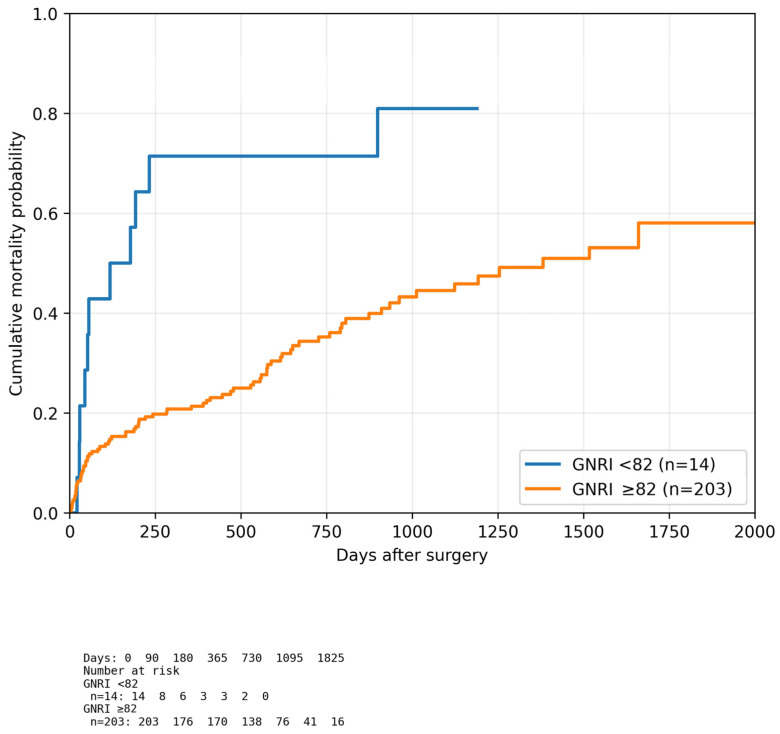
Kaplan–Meier cumulative mortality curves by GNRI high-risk status in the time-to-event cohort (n = 217; log-rank *p* < 0.001). The GNRI < 82 curve contains 14 patients/episodes; the one-year binary GNRI high-risk category contains 13 patients/episodes because one living high-risk patient/episode had <365 days of follow-up and was censored in the binary one-year analysis.

Exploratory early mortality analyses in the full older-adult time-to-event cohort showed a similar direction of effect. Thirty-day mortality was 3/14 (21.4%) in GNRI < 82 versus 13/203 (6.4%) in GNRI ≥82 (Fisher exact *p* = 0.073). Ninety-day mortality was 6/14 (42.9%) versus 27/203 (13.3%), respectively (Fisher exact *p* = 0.010). These exploratory analyses suggest that the GNRI high-risk signal was not limited to late mortality, although the 30-day comparison was underpowered. Kaplan–Meier cumulative mortality curves likewise demonstrated early and persistent separation between the GNRI < 82 and GNRI ≥ 82 groups in the full time-to-event cohort (Figure 2).

### 3.3. Multivariable One-Year and Long-Term Mortality Models

In adjusted one-year logistic regression (Table 3), GNRI high nutritional risk (<82) remained independently associated with one-year mortality (OR 6.43, 95% CI 1.50–27.55; *p* = 0.012). Age and available Charlson-domain weighted comorbidity burden were also independently associated with one-year mortality. ASA III–IV showed a clinically important but statistically borderline association in the one-year logistic model.

The Firth penalized logistic sensitivity analysis attenuated the primary estimate from OR 6.43 to OR 5.51 while preserving directionality and statistical significance, consistent with a stable but imprecise high-risk signal in this sparse subgroup (interpreted further in the Discussion section).

Sensitivity analyses using alternative GNRI operationalisations supported the same direction of association, although effect estimates varied by parameterisation. In the adjusted one-year logistic model, continuous GNRI per 10-point decrease showed OR 1.56 (95% CI 0.95–2.56; *p* = 0.076). When GNRI categories were used, the adjusted high-risk category remained associated with one-year mortality versus the no-risk category (OR 5.68, 95% CI 1.16–27.90; *p* = 0.032), whereas low- and moderate-risk categories were not independently significant. In Cox sensitivity models, GNRI < 82 was associated with long-term mortality (HR 2.31, 95% CI 1.17–4.57; *p* = 0.016), and the high-risk category remained associated with long-term mortality versus no risk (HR 2.35, 95% CI 1.07–5.15; *p* = 0.033). Full results are provided in Supplementary Table S3. In a full-opportunity sensitivity analysis restricted to patients/episodes operated on at least 365 days before the administrative dataset lock date (n = 187; 46 one-year deaths), GNRI < 82 remained associated with one-year mortality (75.0% [9/12] vs. 21.1% [37/175]; adjusted OR 6.21, 95% CI 1.44–26.87; *p* = 0.014). Additional adjustment for operation year did not materially alter the estimate (adjusted OR 6.46, 95% CI 1.50–27.93; *p* = 0.012; Supplementary Table S6).

In the long-term Cox model, age, ASA III–IV, available Charlson-domain weighted comorbidity burden, and lower continuous GNRI were independently associated with all-cause mortality. NLR did not retain independent significance in either the one-year logistic model or the Cox model, and SII did not provide meaningful discrimination in the one-year ROC analysis. Schoenfeld residual assessment did not demonstrate a clinically meaningful proportional hazards violation; an approximate global test based on residual association with log-time was not significant (*p* = 0.612), and individual covariate tests were not significant (all *p* ≥ 0.184).

### 3.4. Model Discrimination, Calibration, and Internal Validation

Among individual predictors, the recorded ACCI field had the highest apparent AUC (0.714), but this variable incorporates age-related prognostic information by construction and was therefore retained as a sensitivity/discrimination variable rather than the primary comorbidity covariate. The recorded ACCI field and reconstructed available Charlson-domain weighted score were moderately correlated but not interchangeable, as shown in Supplementary Table S4. Age, albumin, GNRI, ASA III–IV, and available Charlson-domain weighted burden showed modest individual discrimination; NLR and SII were weaker individual classifiers.

**Figure 3:**
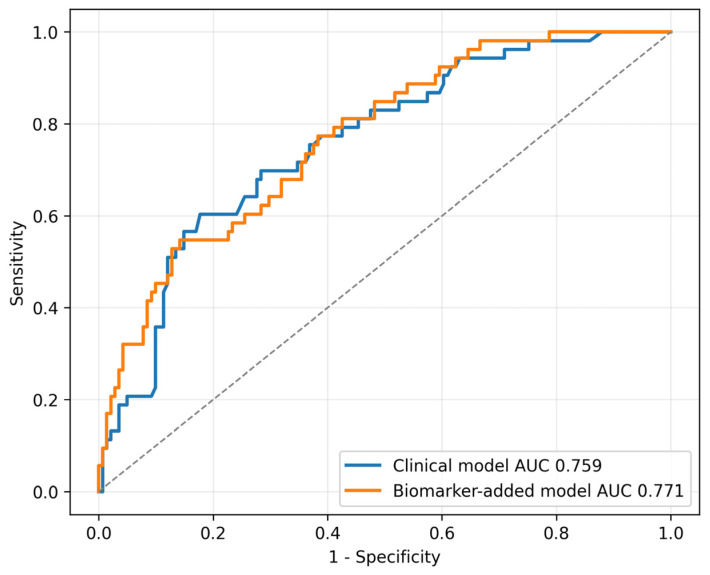
Apparent receiver operating characteristic curves for the clinical and biomarker-added one-year mortality models. These curves describe apparent discrimination in the derivation cohort and should not be interpreted as externally validated incremental clinical utility.

**Figure 4:**
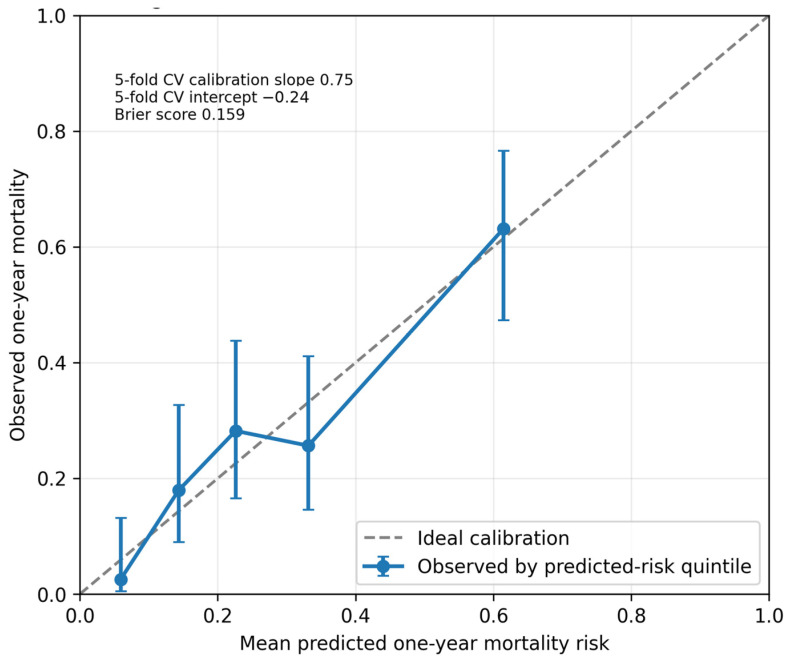
Calibration plot for the biomarker-added one-year mortality model. Observed risk is plotted across predicted-risk quintiles with Wilson 95% confidence intervals.

The clinical model incorporating age, sex, ASA III–IV, and available Charlson-domain weighted burden achieved an apparent AUC of 0.759 (95% CI 0.682–0.833), a five-fold cross-validated AUC of 0.743, and a bootstrap optimism-corrected AUC of 0.738. Adding NLR and GNRI < 82 increased apparent AUC to 0.771 (95% CI 0.699–0.840), while the five-fold cross-validated AUC was 0.735 and the bootstrap optimism-corrected AUC was 0.742. Discrimination results are summarized in Table 4, and the corresponding apparent ROC curves are shown in Figure 3. The cross-validated calibration intercept was −0.24 (95% CI −0.67 to 0.20) and the calibration slope was 0.75 (95% CI 0.45 to 1.05), calculated from pooled out-of-fold predicted probabilities, with the calibration plot shown in Figure 4. The apparent AUC increase after adding NLR and GNRI < 82 was small (+0.012), was not supported by five-fold cross-validation (−0.008), and was minimal after bootstrap optimism correction (+0.004). Therefore, the biomarker-added model was interpreted as showing risk-marker association rather than confirmed incremental predictive utility. A consolidated summary of model diagnostics and sensitivity analyses is provided in Table 5. The first-episode-only analysis, including the corresponding Cox GNRI estimate, is detailed in Supplementary Table S5.

## 4. Discussion

In this single-centre cohort of older patients/fracture episodes undergoing PFN for pertrochanteric hip fracture, severe nutritional risk defined by GNRI < 82 identified a small but highly vulnerable subgroup with markedly elevated one-year mortality. Age, ASA III–IV, available Charlson-domain comorbidity burden, and GNRI were the most consistent mortality signals, whereas NLR, PLR, and SII were weaker after adjustment. Importantly, the addition of GNRI < 82 and NLR produced only limited model-level incremental discrimination after internal validation; therefore, the findings should be interpreted within a risk-marker framework rather than as a ready-to-use prediction tool.

These findings extend previous hip-fracture nutrition literature by showing that a striking severe-GNRI subgroup association can coexist with only modest model-level improvement after internal validation.

The high-risk GNRI signal is biologically plausible and is consistent with the expanding hip-fracture nutrition literature. GNRI integrates serum albumin, body weight, and ideal body weight, thereby capturing nutritional reserve, inflammatory burden, and somatic vulnerability. Systematic reviews and cohort studies have reported that malnutrition and low GNRI are associated with higher postoperative mortality after hip fracture, including early, one-year, and longer-term endpoints [10,11,12,13,14,15,16,17]. Recent work in this area has reinforced that malnutrition is common in older hip-fracture patients and tracks with poorer mobility, more complications, and worse survival, while also showing that nutrition-related indices can behave differently across cohorts, age strata, and endpoints; accordingly, GNRI is best interpreted as a nutrition-related risk marker rather than a stand-alone prognostic instrument [30,31,32,33]. Consistent with this framing, in a cohort of 548 patients aged 80 years and older undergoing surgery for proximal femur fracture, GNRI was not an independent predictor of postoperative morbidity, whereas serum albumin and the Charlson Comorbidity Index showed modestly better discrimination (areas under the curve 0.647 and 0.648, respectively) [32]. Older patients with severe nutritional compromise may be less able to tolerate the catabolic stress of fracture, anesthesia, postoperative inflammation, immobilization, and rehabilitation delay.

However, the GNRI < 82 subgroup contained only 13 patients/episodes in the one-year binary cohort. This sparse-data structure can inflate maximum-likelihood odds ratios and produce unstable point estimates. The Firth penalized logistic sensitivity analysis attenuated the primary estimate from OR 6.43 to OR 5.51 while preserving directionality and statistical significance. This supports the interpretation of GNRI < 82 as a clinically relevant high-risk signal, but the wide confidence interval and sparse subgroup size indicate that the magnitude of effect remains imprecise. The result should therefore be viewed as hypothesis-strengthening and clinically alerting rather than as a definitive effect estimate. Clinically, the confidence interval should be interpreted as evidence that the direction of association is likely adverse, but that the true magnitude may range from a modest to a very large excess risk. Therefore, the absolute 76.9% one-year mortality observed in the GNRI < 82 subgroup should be treated as a warning signal for clinical vulnerability rather than as a stable subgroup-specific risk estimate. This limitation is structural and cannot be fully resolved by penalized regression in the absence of a larger externally validated high-risk cohort.

Comorbidity burden also remained important. The recorded institutional ACCI field showed the highest individual AUC, but its use in the primary adjusted model would risk double-counting age because ACCI incorporates age-related prognostic information by construction. We therefore reconstructed an available Charlson-domain weighted burden score from dataset fields and modelled age separately. This approach improved transparency and avoided age duplication, but it is not equivalent to a fully validated Charlson index because several Charlson domains and severity gradations were unavailable. Consequently, the reconstructed score should be interpreted as a pragmatic comorbidity-burden measure. The exploratory ACCI sensitivity model achieved higher discrimination than the primary clinical model, but this was expected because the recorded field incorporated age-related risk information in addition to comorbidity burden. Since age was modelled explicitly as a separate covariate, using ACCI as the primary covariate would have risked partial duplication of the age effect. For this reason, the reconstructed available-domain score was selected for the primary model and the recorded ACCI field was presented as an exploratory sensitivity/discrimination analysis.

Dementia/Alzheimer disease showed a non-significant but clinically relevant imbalance in Table 1. It was roughly twice as frequent among one-year non-survivors as among survivors (24.5% versus 13.5%; *p* = 0.065). Cognitive impairment may interact with nutritional vulnerability through reduced oral intake, delirium susceptibility, immobility, poorer rehabilitation participation, and greater dependence on caregiver or institutional support. Several of these pathways act directly on the components of the GNRI, because reduced intake and unintentional weight loss lower both serum albumin and body weight, while shared underlying frailty and systemic inflammation link cognitive and nutritional decline through common mechanisms. Low GNRI and dementia are therefore likely to behave as partially convergent markers of a frail, high-risk phenotype rather than as fully independent risk factors, which suggests that patients flagged by a low GNRI should also be screened for cognitive impairment and delirium risk. Because dementia was represented within the available Charlson-domain burden and events were limited, it was not modelled separately; however, the observed trend supports interpreting GNRI within a broader frailty and cognitive-vulnerability context rather than as an isolated nutrition construct.

Sex differences also warrant cautious interpretation. Female sex was less frequent among one-year non-survivors in unadjusted comparisons and showed a borderline protective direction in adjusted logistic and Cox models. This may reflect sex-related differences in baseline survival, comorbidity profile, frailty phenotype, or residual confounding, but the study was not powered to explore sex-specific mechanisms or interactions. Therefore, sex was retained as an adjustment covariate rather than interpreted as a primary biological finding.

In contrast to the strong subgroup signal for severe nutritional risk, CBC-derived inflammatory indices (NLR, PLR, SII) did not provide clinically meaningful incremental discrimination after internal validation. NLR, PLR, and SII were numerically higher among one-year non-survivors, but only NLR was retained as a prespecified covariate and it did not independently predict one-year or long-term mortality after adjustment. This does not negate the biological relevance of inflammation after hip fracture; rather, it suggests that, in this dataset, nutritional reserve and clinical comorbidity burden were more robust prognostic signals than systemic inflammatory ratios alone. This interpretation is consistent with a heterogeneous literature in which NLR and SII associations vary according to timing of measurement, cut-off selection, adjustment strategy, and outcome horizon [18,19,20,21,22]. The limited incremental value of CBC-derived ratios in the present cohort is consistent with broader concerns that individual inflammatory or metabolic markers may show apparent associations without robust, transportable discrimination, and that albumin-based composites and large marker-comparison studies are better read as context-dependent markers [34,35,36]. For example, in a comparison of 27 immune-inflammatory-metabolic markers in 1273 hip-fracture patients, individual indices showed only modest and context-dependent discrimination [35]; and in a validation cohort of 1113 patients, although NLR was independently associated with mortality and showed a clear gradient across rising categories (mortality 26.2%, 36.5%, and 54.4% for values below 5, 5 to 10, and above 10, respectively), its stand-alone discrimination remained limited (area under the curve 0.614) [36]. The retrospective timing of laboratory sampling is particularly relevant for interpreting albumin-based and CBC-derived markers. In older patients with hip fracture, the acute-phase response may evolve rapidly after injury, potentially lowering albumin and increasing neutrophil-dominant inflammatory ratios before surgery. This may introduce differential measurement bias: NLR, PLR, and SII may be more sensitive to acute inflammatory timing, whereas GNRI combines albumin with anthropometric reserve and may therefore reflect both acute illness and pre-existing nutritional vulnerability. Because standardized timestamps separating admission from immediately preoperative samples were not consistently available, the present findings should be interpreted as associations with routinely available preoperative laboratory values rather than precisely timed biological measurements. This timing heterogeneity may partly explain why acute inflammatory indices lost significance after adjustment. Because each sample could have been drawn at a variable interval after injury, the same numerical NLR, PLR, or SII value may correspond to different points along the evolving acute-phase response, so that any true prognostic signal carried by these dynamic markers would be partially diluted by non-standardized sampling time. Nutritional reserve as captured by GNRI is comparatively more stable over short intervals and would be expected to be less degraded by this variation, which is consistent with the more robust GNRI signal observed here.

The biomarker-added model achieved reasonable apparent discrimination, but internal validation suggested optimism: AUC declined from 0.771 to 0.735 in five-fold cross-validation and to 0.742 after bootstrap optimism correction. The cross-validated calibration slope of 0.75 similarly suggests mild optimism, which is expected in a modest single-centre dataset with 53 one-year events and a rare high-risk subgroup. A calibration slope below 1 indicates that predictions may be too extreme when transported to new patients, with low predicted risks tending to be too low and high predicted risks tending to be too high. In the calibration plot, observed event rates broadly increased across predicted-risk quintiles, but the confidence intervals were wide and did not support precise patient-level risk estimation. Therefore, the model should be used only to describe cohort-level marker behaviour, not to assign exact individual mortality probabilities or treatment thresholds. Clinically, routine GNRI calculation could complement established bedside risk assessment by flagging patients for early orthogeriatric and nutritional assessment, intensified perioperative optimization, discharge planning, and post-discharge surveillance; it should not delay urgent fracture fixation or replace multidisciplinary clinical judgement [6,37,38,39,40].

The present analysis should also be distinguished from studies addressing mechanical optimization of cephalomedullary fixation. In selected osteoporotic trochanteric fractures, cement augmentation of a perforated blade has been proposed to improve femoral-head purchase and reduce cut-out; a recent retrospective comparison found no overall reduction in varus deformity but suggested that prevention of cut-out in selected subgroups requires further study [41]. Such surgical modifications may reduce mechanical failure or reoperation risk, but whether they modify systemic mortality driven by frailty, malnutrition, and comorbidity remains uncertain. Cement augmentation was not evaluated in the present Talon DistalFix PFN cohort; therefore, our mortality estimates should not be interpreted as evidence for or against augmentation.

This study has several limitations. Its retrospective single-centre design limits generalisability and introduces risk of residual confounding and information bias. Cause-specific mortality, frailty scores, prefracture mobility, validated cognitive status severity, fracture classification, time to surgery, anesthesia type, postoperative complications, sarcopenia measures, and nutritional interventions were not comprehensively available. Although these domains were unavailable here, recent hip-fracture literature indicates that prefracture mobility, frailty, sarcopenia, rehabilitation pathway, and postoperative course are closely linked to recovery and survival, which bounds the conclusions that the present adjusted estimates can support [23,24]. In particular, surgical timing is an established determinant of postoperative mortality that could not be incorporated [42,43], and validated frailty measures, likewise unavailable, carry strong prognostic value in this population [44]. Accordingly, the term “preoperative” refers to the timing of the evaluated risk markers and should not be interpreted as implying adjustment for intraoperative variables or detailed perioperative care processes. Because AO/OTA or Evans fracture subclassification was not consistently available, pertrochanteric/intertrochanteric terminology was used as an anatomic treatment-cohort descriptor rather than as a fracture-severity adjustment variable. Albumin is an acute-phase reactant and may reflect inflammation as well as nutritional status, although it remains an established prognostic nutritional marker after hip-fracture surgery [45]. The exact timing and selection rule for preoperative laboratory values may introduce measurement heterogeneity because standardized admission-versus-immediately preoperative time stamps were not available in the analytic file. Height and weight were retrieved from routine clinical fields, and potential estimation, rounding, or use of previously recorded values may have introduced GNRI misclassification, particularly for patients near the category thresholds of 82, 92, and 98. Such misclassification would most likely attenuate true associations if nondifferential, although its direction cannot be verified retrospectively. Vital status was ascertained using hospital mortality records and the national death registry; however, cause-specific mortality and registry-source coding were not available in the analytic file. The reconstructed Charlson-domain burden relied on available comorbidity fields and did not capture all original Charlson domains or severity categories. In addition, the primary unit of analysis was the surgical/fracture episode; although only two identifiers contributed repeated episodes and first-episode-only sensitivity analysis did not materially change the findings, residual within-patient dependence cannot be fully excluded. The GNRI < 82 subgroup was small, and although penalized regression supported the association, the effect size remains imprecise. Finally, internal validation cannot substitute for external validation in an independent multicentre cohort.

## 5. Conclusions

In older adults undergoing PFN for pertrochanteric hip fracture, GNRI < 82 identified a small subgroup with high observed one-year mortality. Age, ASA III–IV, available Charlson-domain comorbidity burden, and GNRI were more consistent mortality signals than CBC-derived inflammatory indices. Routine GNRI calculation may help flag patients for early orthogeriatric and nutritional assessment; however, the sparse GNRI < 82 subgroup, modest incremental discrimination after internal validation, and single-centre retrospective design mean that larger prospective multicentre validation is required before GNRI-based thresholds are incorporated into formal risk-stratification pathways.

## Figures and Tables

**Table 1:** Baseline characteristics of the one-year evaluable cohort stratified by one-year mortality.

Variable	Total (n = 194)	Survived 1 Year (n = 141)	Died ≤ 1 Year (n = 53)	*p*-Value
Age, years	82.0 (74.0–86.0)	79.0 (74.0–86.0)	85.0 (80.0–90.0)	<0.001
Body mass index, kg/m^2^	26.7 (23.3–30.1)	26.8 (23.0–30.4)	26.6 (24.0–28.8)	0.575
Albumin, g/L	35.5 (32.3–38.4)	35.9 (33.3–38.6)	33.0 (28.5–37.7)	0.002
GNRI	93.8 (89.0–98.8)	94.9 (91.0–99.0)	90.4 (84.1–97.8)	0.003
NLR	5.00 (2.92–7.27)	4.70 (2.83–6.86)	5.41 (3.65–8.04)	0.177
PLR	150.8 (107.3–210.5)	149.2 (106.5–206.7)	155.9 (113.3–219.1)	0.535
SII	1047 (609–1795)	992 (609–1780)	1178 (625–1989)	0.422
Recorded ACCI	5.0 (4.0–6.0)	4.0 (4.0–5.0)	6.0 (5.0–7.0)	<0.001
Available Charlson-domain weighted score	2.0 (1.0–2.0)	1.0 (1.0–2.0)	2.0 (1.0–3.0)	0.004
Female sex	127 (65.5)	99 (70.2)	28 (52.8)	0.023
ASA III–IV	130 (67.0)	85 (60.3)	45 (84.9)	0.001
Hypertension	133 (68.6)	98 (69.5)	35 (66.0)	0.643
Diabetes mellitus	77 (39.7)	52 (36.9)	25 (47.2)	0.192
Coronary artery disease	69 (35.6)	49 (34.8)	20 (37.7)	0.699
COPD/asthma	54 (27.8)	37 (26.2)	17 (32.1)	0.419
Dementia/Alzheimer disease	32 (16.5)	19 (13.5)	13 (24.5)	0.065
Congestive heart failure	14 (7.2)	10 (7.1)	4 (7.5)	1.000
Chronic kidney disease	8 (4.1)	5 (3.5)	3 (5.7)	0.686
Cancer history	10 (5.2)	6 (4.3)	4 (7.5)	0.465
Prior stroke/CVA	40 (20.6)	27 (19.1)	13 (24.5)	0.409

Data are median (IQR) or n (%). ACCI = recorded institutional age-adjusted Charlson Comorbidity Index; GNRI = Geriatric Nutritional Risk Index; NLR = neutrophil-to-lymphocyte ratio; PLR = platelet-to-lymphocyte ratio; SII = systemic immune-inflammation index; CVA = cerebrovascular accident; ASA = American Society of Anesthesiologists physical status; COPD = chronic obstructive pulmonary disease The available Charlson-domain weighted score is not a validated full Charlson Comorbidity Index.

**Table 2:** GNRI nutritional-risk categories in the full time-to-event and one-year evaluable cohorts.

GNRI Category	Full Time-to-Event Cohort n = 217	One-Year Evaluable Cohort n = 194	One-Year Deaths	One-Year Mortality (%)	OR vs. no Risk (95% CI)	*p*
No risk (≥98)	61	55	12	21.8	Reference	-
Low risk (92 to <98)	69	64	10	15.6	0.66 (0.26–1.68)	0.479
Moderate risk (82 to <92)	73	62	21	33.9	1.84 (0.80–4.20)	0.157
High risk (<82)	14	13	10	76.9	11.94 (2.83–50.42)	<0.001

The GNRI < 82 category included 14 patients/episodes in the full time-to-event cohort and 13 in the one-year evaluable binary cohort because one living high-risk patient/episode had <365 days of follow-up and was censored in survival analyses. ORs are unadjusted and calculated versus the GNRI no-risk reference category. GNRI = Geriatric Nutritional Risk Index.

**Table 3:** Adjusted predictors of one-year and long-term mortality.

Variable	One-Year OR (95% CI)	*p* Value	Long-Term HR (95% CI)	*p* Value
ASA III–IV	2.11 (0.85–5.20)	0.106	2.00 (1.16–3.43)	0.012
Age (per 5-year increase)	1.34 (1.04–1.73)	0.024	1.22 (1.05–1.42)	0.008
Female sex	0.53 (0.25–1.12)	0.096	0.66 (0.43–1.02)	0.059
GNRI (per 10-point decrease)	—	—	1.38 (1.01–1.87)	0.042
GNRI high nutritional risk (<82)	6.43 (1.50–27.55)	0.012	—	—
NLR (per doubling)	1.30 (0.91–1.87)	0.153	1.13 (0.90–1.41)	0.306
Available Charlson-domain weighted score (per point)	1.51 (1.15–1.99)	0.003	1.20 (1.04–1.38)	0.011

The one-year logistic model used GNRI < 82 as the primary nutritional-risk variable because the one-year binary mortality signal was concentrated in the severe nutritional-risk subgroup. The Cox model used continuous GNRI per 10-point decrease to preserve information across the full time-to-event cohort and avoid excessive reliance on the sparse GNRI < 82 subgroup. Parallel sensitivity analyses showed consistent directionality: continuous GNRI in the one-year logistic model yielded OR 1.56 per 10-point decrease (95% CI 0.95–2.56; *p* = 0.076), whereas GNRI < 82 in the Cox model yielded HR 2.31 (95% CI 1.17–4.57; *p* = 0.016). Full alternative GNRI operationalisations are presented in Supplementary Table S3. OR = odds ratio; HR = hazard ratio.

**Table 4:** Discrimination and internal validation for one-year mortality.

Predictor/Model	Apparent AUC (95% CI)	5-Fold CV AUC	Bootstrap Optimism-Corrected AUC
Age	0.663 (0.573–0.752)	—	—
Recorded ACCI	0.714 (0.643–0.791)	—	—
Available Charlson-domain weighted score	0.629 (0.544–0.711)	—	—
ASA III–IV	0.623 (0.558–0.685)	—	—
Albumin	0.641 (0.551–0.734)	—	—
GNRI (lower values = higher risk)	0.637 (0.546–0.735)	—	—
NLR (log2)	0.563 (0.467–0.646)	—	—
SII (log2)	0.538 (0.432–0.622)	—	—
Clinical model: age + sex + ASA + Charlson-domain burden	0.759 (0.682–0.833)	0.743	0.738
Biomarker-added model: clinical model + NLR + GNRI < 82	0.771 (0.699–0.840)	0.735	0.742
ACCI sensitivity model: age + sex + ASA + recorded ACCI + NLR + GNRI < 82	0.799 (0.728–0.863)	0.763	0.770

AUC = area under the receiver operating characteristic curve; CV = cross-validation. Cross-validation and optimism-corrected AUC are shown for multivariable models only. The ACCI sensitivity model is presented as exploratory because the recorded ACCI field incorporates age by construction.

**Table 5:** Summary of model diagnostics and sensitivity analyses.

Analysis	Estimate/Result	Interpretation
Maximum-likelihood adjusted logistic model, GNRI < 82	OR 6.43 (95% CI 1.50–27.55), *p* = 0.012	Primary one-year model
Firth penalized logistic model, GNRI < 82	OR 5.51 (95% CI 1.34–22.61), *p* = 0.018	Sparse-data sensitivity; association preserved
Continuous GNRI logistic sensitivity	OR 1.56 per 10-point decrease (95% CI 0.95–2.56), *p* = 0.076	Direction consistent but not statistically significant
GNRI category logistic sensitivity, high risk vs. no risk	OR 5.68 (95% CI 1.16–27.90), *p* = 0.032	High-risk category drives the signal
Cox sensitivity, GNRI < 82	HR 2.31 (95% CI 1.17–4.57), *p* = 0.016	Long-term mortality signal preserved
Cox category sensitivity, high risk vs. no risk	HR 2.35 (95% CI 1.07–5.15), *p* = 0.033	High-risk category associated with long-term mortality
Kaplan–Meier-estimated one-year mortality	24.6% overall; 71.4% for GNRI < 82; 21.4% for GNRI ≥ 82	Consistent with binary one-year endpoint
Full-opportunity sensitivity, GNRI < 82	OR 6.21 (95% CI 1.44–26.87), *p* = 0.014	Full 365-day follow-up opportunity; association preserved
Full-opportunity + operation-year sensitivity, GNRI < 82	OR 6.46 (95% CI 1.50–27.93), *p* = 0.012	Calendar-time adjustment did not materially alter estimate
Recorded ACCI vs. available Charlson-domain score	Spearman rho = 0.607 in ≥65 cohort, *p* < 0.001	Related but not interchangeable
First-episode-only sensitivity	GNRI < 82 OR 6.35 (95% CI 1.48–27.29), *p* = 0.013	Repeated episodes did not materially alter primary signal
Biomarker-added model internal validation	Apparent AUC 0.771; CV AUC 0.735; optimism-corrected AUC 0.742	Limited incremental predictive utility after internal validation
Cross-validated calibration slope	0.75 (95% CI 0.45 to 1.05)	Mild optimism; external validation required
Early mortality by GNRI < 82	30-day: 21.4% vs. 6.4% (*p* = 0.073); 90-day: 42.9% vs. 13.3% (*p* = 0.010)	Directionally higher early mortality

PH = proportional hazards. Alternative GNRI operationalisations, comorbidity-correlation analyses, and first-episode-only sensitivity details are provided in Supplementary Tables S3–S6.

## Data Availability

The data presented in this study are available from the corresponding author upon reasonable request. The data are not publicly available due to institutional and ethical restrictions concerning patient-level clinical data.

## Supplementary Materials

The following supporting information can be downloaded at: https://www.mdpi.com/article/10.3390/jcm15145400/s1, Table S1: Mapping of available comorbidity fields to the available Charlson-domain weighted burden score; Table S2: Comparison of one-year evaluable patients/episodes and living patients/episodes censored before one year; Table S3: Sensitivity analyses using alternative GNRI operationalisations; Table S4: Relationship between recorded ACCI and available Charlson-domain weighted comorbidity burden; Table S5: First-episode-only sensitivity analysis for repeated identifiers; Table S6: Full-opportunity and calendar-year-adjusted sensitivity analyses for one-year mortality.
