# Supplementary material for: Preoperative Geriatric Nutritional Risk Index (GNRI) and Comorbidity Burden as Mortality Risk Markers After Proximal Femoral Nailing in Older Patients with Pertrochanteric Hip Fractures
Source: J Clin Med. 2026 Jul 9;15(14):5400. doi: 10.3390/jcm15145400 (PMC13410370; doi:10.3390/jcm15145400)
Supplement: Supplementary file 1 [file jcm-15-05400-s001.zip › Supplementary Table S1.pdf]

Supplementary Table S1. Mapping of available comorbidity fields to the available Charlson-domain weighted burden score

Dataset field	Mapped Charlson-compatible domain	Assigned weight	Included in score
Diabetes mellitus	Diabetes without recorded end-organ damage	1	Yes
Coronary artery disease	Myocardial infarction/ischemic heart disease proxy	1	Yes
COPD/asthma	Chronic pulmonary disease	1	Yes
Dementia/Alzheimer disease	Dementia	1	Yes
Congestive heart failure	Congestive heart failure	1	Yes
Chronic kidney disease	Moderate/severe renal disease proxy	2	Yes
Cancer history	Any tumour/malignancy proxy	2	Yes
Prior stroke/CVA	Cerebrovascular disease	1	Yes
Cirrhosis	Moderate/severe liver disease proxy	3	Yes
Hypertension	Not an original Charlson domain	0	No
Atrial fibrillation	Not an original Charlson domain	0	No
Parkinson disease	Not an original Charlson domain	0	No
Hypo/hyperthyroidism	Not an original Charlson domain	0	No
Behçet disease	Not an original Charlson domain	0	No
Pulmonary embolism	Not an original Charlson domain	0	No

The available Charlson-domain weighted burden score is not a full Charlson Comorbidity Index. It is a pragmatic age-separated comorbidity-burden variable reconstructed from available fields to reduce age double-counting in adjusted models. Hypertension, atrial fibrillation, Parkinson disease, thyroid disease, Behçet disease, and pulmonary embolism were not scored because they are not original Charlson domains.
