# Supplementary material for: Preoperative Geriatric Nutritional Risk Index (GNRI) and Comorbidity Burden as Mortality Risk Markers After Proximal Femoral Nailing in Older Patients with Pertrochanteric Hip Fractures
Source: J Clin Med. 2026 Jul 9;15(14):5400. doi: 10.3390/jcm15145400 (PMC13410370; doi:10.3390/jcm15145400)
Supplement: Supplementary file 1 [file jcm-15-05400-s001.zip › Supplementary Table S2.pdf]

Supplementary Table S2. Comparison of one-year evaluable patients/episodes and living patients/episodes censored before one year

Variable	One-year evaluable n=194	Living censored <365 days n=23	p
Age, years	82.0 (74.0-86.0)	80.0 (74.5-85.5)	0.872
BMI, kg/m²	26.7 (23.3-30.1)	26.2 (22.7-28.0)	0.315
Albumin, g/L	35.5 (32.3-38.4)	35.0 (32.5-38.2)	0.862
GNRI	93.8 (89.0-98.8)	91.7 (90.1-98.7)	0.703
GNRI <82	13 (6.7%)	1 (4.3%)	1.000
NLR	5.00 (2.92-7.27)	4.35 (3.11-6.09)	0.379
PLR	150.8 (107.3-210.5)	143.1 (117.3-165.9)	0.450
SII	1047 (609-1795)	941 (570-1311)	0.280
Recorded ACCI	5.0 (4.0-6.0)	4.0 (4.0-6.0)	0.567
Available Charlson-domain score	2.0 (1.0-2.0)	1.0 (1.0-2.0)	0.936
Female sex	127 (65.5%)	14 (60.9%)	0.662
ASA III-IV	130 (67.0%)	19 (82.6%)	0.127

Data are median (IQR) or n (%). Continuous variables were compared using Mann-Whitney U tests. Categorical variables were compared using chi-square or Fisher exact tests, as appropriate.
