# Supplementary material for: Preoperative Geriatric Nutritional Risk Index (GNRI) and Comorbidity Burden as Mortality Risk Markers After Proximal Femoral Nailing in Older Patients with Pertrochanteric Hip Fractures
Source: J Clin Med. 2026 Jul 9;15(14):5400. doi: 10.3390/jcm15145400 (PMC13410370; doi:10.3390/jcm15145400)
Supplement: Supplementary file 1 [file jcm-15-05400-s001.zip › Supplementary Table S3.pdf]

Supplementary Table S3. Sensitivity analyses using alternative GNRI operationalisations

Analysis	Model/contrast	Estimate (95% CI)	p
One-year logistic	GNRI per 10-point decrease	OR 1.56 (0.95-2.56)	0.076
One-year logistic	GNRI low risk vs no risk	OR 0.43 (0.15-1.24)	0.118
One-year logistic	GNRI moderate risk vs no risk	OR 1.16 (0.46-2.91)	0.749
One-year logistic	GNRI high risk vs no risk	OR 5.68 (1.16-27.90)	0.032
Cox long-term mortality	GNRI <82 vs ≥82	HR 2.31 (1.17-4.57)	0.016
Cox long-term mortality	GNRI low risk vs no risk	HR 0.82 (0.46-1.46)	0.496
Cox long-term mortality	GNRI moderate risk vs no risk	HR 1.12 (0.64-1.96)	0.688
Cox long-term mortality	GNRI high risk vs no risk	HR 2.35 (1.07-5.15)	0.033

Models were adjusted for age, sex, ASA III-IV, NLR, and available Charlson-domain weighted comorbidity burden. Logistic models used the one-year evaluable cohort; Cox models used the full time-to-event cohort.
