# Supplementary material for: Preoperative Geriatric Nutritional Risk Index (GNRI) and Comorbidity Burden as Mortality Risk Markers After Proximal Femoral Nailing in Older Patients with Pertrochanteric Hip Fractures
Source: J Clin Med. 2026 Jul 9;15(14):5400. doi: 10.3390/jcm15145400 (PMC13410370; doi:10.3390/jcm15145400)
Supplement: Supplementary file 1 [file jcm-15-05400-s001.zip › Supplementary Table S4.pdf]

Supplementary Table S4. Relationship between recorded ACCI and available Charlson-domain weighted comorbidity burden

Cohort	n	Spearman rho	p
Complete adult analytic records	242	0.643	<0.001
≥65 time-to-event cohort	217	0.607	<0.001
One-year evaluable cohort	194	0.638	<0.001

The recorded ACCI field and reconstructed available Charlson-domain weighted burden score were related but not interchangeable. The reconstructed score was used in primary models to avoid double-counting age.
