# Supplementary material for: Preoperative Geriatric Nutritional Risk Index (GNRI) and Comorbidity Burden as Mortality Risk Markers After Proximal Femoral Nailing in Older Patients with Pertrochanteric Hip Fractures
Source: J Clin Med. 2026 Jul 9;15(14):5400. doi: 10.3390/jcm15145400 (PMC13410370; doi:10.3390/jcm15145400)
Supplement: Supplementary file 1 [file jcm-15-05400-s001.zip › Supplementary Table S5.pdf]

Supplementary Table S5. First-episode-only sensitivity analysis for repeated identifiers

Analysis	Result
Older unique first-episode cohort	n=215
One-year evaluable first-episode cohort	n=192
One-year deaths	n=53
Primary logistic model: GNRI <82	OR 6.35 (95% CI 1.48-27.29), p=0.013
Cox model: continuous GNRI per 10-point decrease	HR 1.37 (95% CI 1.01-1.86), p=0.043

Two identifiers contributed two eligible PFN episodes each in the complete analytic dataset. Retaining only the first eligible episode did not materially alter the primary GNRI signal.
