# Supplementary material for: Preoperative Geriatric Nutritional Risk Index (GNRI) and Comorbidity Burden as Mortality Risk Markers After Proximal Femoral Nailing in Older Patients with Pertrochanteric Hip Fractures
Source: J Clin Med. 2026 Jul 9;15(14):5400. doi: 10.3390/jcm15145400 (PMC13410370; doi:10.3390/jcm15145400)
Supplement: Supplementary file 1 [file jcm-15-05400-s001.zip › Supplementary Table S6.pdf]

Supplementary Table S6. Full-opportunity and calendar-year-adjusted sensitivity analyses for one-year mortality

Sensitivity model	Cohort / outcome	GNRI <82 estimate	Calendar-time term	Interpretation
Full-opportunity cohort	n=187; 46 one-year deaths	OR 6.21 (95% CI 1.44-26.87), p=0.014	Not included	Association preserved when all included patients/episodes had full 365-day follow-up opportunity
Full-opportunity + operation year	n=187; 46 one-year deaths	OR 6.46 (95% CI 1.50-27.93), p=0.012	Operation year OR 0.95 (95% CI 0.84-1.07), p=0.378	Calendar-year adjustment did not materially alter the GNRI <82 estimate
One-year evaluable + operation year	n=194; 53 one-year deaths	OR 6.44 (95% CI 1.50-27.63), p=0.012	Operation year OR 1.00 (95% CI 0.89-1.12), p=0.973	No material evidence of calendar-time confounding

All models were adjusted for age, sex, ASA III-IV, NLR (log2), and available Charlson-domain weighted comorbidity burden. The full-opportunity cohort was restricted to patients/episodes operated on at least 365 days before the administrative dataset lock date of 19 February 2026.
